# Open questions: why are babies rarely born with cancer?

**DOI:** 10.1186/s12915-018-0601-9

**Published:** 2018-11-01

**Authors:** Michelle Monje

**Affiliations:** Departments of Neurology and Pediatrics, Stanford University, 265 Campus Drive, Stanford, CA USA

## Abstract

Childhood cancer is fundamentally a disease of dysregulated development. Why does it rarely occur during the fetal period, a time of enormous growth and development?

Cancer is the leading cause of disease-related morbidity and mortality during childhood. Fundamentally, childhood cancers are diseases of dysregulated development. So why does cancer rarely form during the most intense period of human development? The prenatal period is a time of enormous growth, with the length of a human embryo increasing from 3 mm at 3-weeks gestation to 90 mm at 12-weeks gestation, and the fetus growing to ~ 500 mm by birth. This represents a ~ 170-fold increase in size through a massive expansion of cellular proliferation during prenatal development. And yet, congenital cancer—defined as cancer emerging during the prenatal period up to the first 3 months of postnatal life—is rare, accounting for only 1–2% of all pediatric cancers with a prevalence of one case in 12,500–27,500 live births [1]. By the middle of the first year of life, however, this period of exemption wanes and cancer incidence sharply increases.

Is congenital cancer rare because time and environmental exposure are required to accumulate the mutations that drive malignancy? Several lines of evidence suggest that the answer is no. First, pediatric cancers exhibit a low mutational burden relative to adult cancers [2, 3], so low that in fact a significant proportion (10%) of childhood cancers exhibit no mutations at all [2, 3]. These genomically silent childhood cancers appear to be epigenetically driven [4], but how these cancer cells arrive at a malignant state remains to be fully elucidated. This low mutational burden suggests that childhood cancer initiation does not necessarily require the accumulation of multiple oncogenic mutations. Second, in several examples of childhood cancer the driver mutation occurs prenatally, but the cancer does not emerge until a later timepoint in childhood development. For example, leukemogenic mutations can be retrospectively identified in the neonatal blood spots of young children with B-cell acute lymphoblastic leukemia (B-ALL), indicating that the key mutation was present prenatally but cancer initiation was delayed until later in infancy [5]. Thus, in the prenatal period the critical mutation may be present, cellular proliferation is at a peak, the tissue microenvironment is rich with growth signals, and yet tumors only rarely occur.

Is a protective mechanism at play? Do cells in the prenatal and perinatal period of development exist in a cellular state less permissive to transformation? If this speculation is correct, understanding the manner in which fetal cells typically guard against malignancy could elucidate novel therapeutic strategies applicable to cancer prevention and therapy at all ages.
